# A Multi-Facility, Randomized, Comparative Study Examining the Efficacy of Biliary Reconstruction Under a Surgical Microscope in Living Donor Liver Transplantation

**DOI:** 10.29337/ijsp.151

**Published:** 2021-07-28

**Authors:** Akihiko Soyama, Tomoharu Yoshizumi, Mitsuhisa Takatsuki, Noboru Harada, Takeo Toshima, Shinichiro Ono, Takanobu Hara, Hajime Matsushima, Takayuki Tanaka, Hajime Imamura, Tomohiko Adachi, Masaaki Hidaka, Susumu Eguchi

**Affiliations:** 1 Department of Surgery, Nagasaki University Graduate School of Biomedical Sciences, JP; 2 Department of Surgery and Science, Graduate School of Medical Sciences, Kyushu University, JP; 3 Department of Digestive and General Surgery University of the Ryukyus, JP

**Keywords:** liver transplantation, biliary stricture, bile leakage, biliary reconstruction

## Abstract

**Introduction:**

Postoperative biliary complications in living donor liver transplantation are often difficult to treat, and if treatment is not successful, the patient’s QOL is significantly reduced. The frequency of postoperative biliary complications is reported to be higher than that of deceased donor transplantation. In 2013, Lin et al. reported that while biliary reconstruction has traditionally used a surgical surgical loupe (2.5x–4.5x), biliary reconstruction using a surgical microscope (5x–15x) can reduce the incidence of complications. The objective of this study is to clarify the efficacy of biliary reconstruction using surgical microscope in living donor liver transplantation by a multi-facility, randomized comparative study.

**Methods and analysis:**

It is an open-label randomized controlled study in which target patients who meet the registration requirements are randomly allocated to a surgical loupe group and a microscopy group after obtaining their consent (Ratio 1:1). The primary endpoint is an incidence of biliary complications (bile leakage and anastomotic biliary stricture) with Clavien-Dindo class III or higher within 52 weeks following surgery. The secondary endpoint is length of time required for biliary reconstruction using a surgical microscope.

**Ethics and dissemination:**

This study protocol was approved by the institutional review board of Nagasaki University Hospital (No. 20122102-2). The study is registered in UMIN-CTR as UMIN000042011. Written informed consent will be obtained from all participants. The results will be published in a peer-reviewed journal and will be presented at medical meetings.

**Highlights:**

- Postoperative biliary complications in living donor liver transplantation are often difficult to treat.
- Lower incidence of biliary complication following biliary reconstruction using a surgical microscope has been reported.
- Facilities those use a surgical microscope for biliary reconstruction are limited.
- The first study to investigate the efficacy of surgical microscope for biliary construction in liver transplantation by randomized controlled trial.

## Background

Postoperative biliary complications in living donor liver transplantation are often difficult to treat, and if treatment is not successful, the patient’s QOL is significantly reduced, with some cases resulting in cholestatic cirrhosis, which is referred to as the “Achilles’ heel of living donor liver transplantation” [1]. In actual clinical practice, while a surgical loupe (enlargement ratio: 2.5x–4.5x) is used to perform bile duct to bile duct anastomosis, in the case of liver transplantation using a whole liver graft such as brain-dead donor liver transplantation, the diameter of the reconstructed biliary duct may have wider orifice, with the frequency of biliary complications reported to be approximately 10–15% [2,3,4]. On the other hand, in liver transplantation using a partial liver graft such as living donor liver transplantation, the frequency of postoperative biliary complications is reported to be higher than that of deceased donor transplantation approximately at 15–30% because the diameter of the reconstructed biliary duct is small, short and sometimes multiple lumens [5,6,7].

A surgical microscope (5x–15x) is used for hepatic artery anastomosis with a diameter of 2 to 3 mm in living donor liver transplantation; however, not many facilities use them for biliary reconstruction. In 2013, Lin et al. reported a retrospective examination at a single facility that while biliary reconstruction has traditionally used a surgical loupe (2.5x–4.5x), biliary reconstruction using a surgical microscope (5x–15x) can reduce the incidence of complications to the 6% range [8].

## Objective

The objective of this study is to clarify the efficacy of biliary reconstruction using surgical microscopes in living donor liver transplantation, in which good treatment results have been reported, by conducting a multi-facility, randomized comparative study.

## Expected results

If this study proves the usefulness of biliary reconstruction using a microscope, the technique will become one standard technique, with expectations of low incidence of biliary complications following liver transplantation as well as improved patient QOL by performing this technique.

## Study design

It is an open-label randomized controlled study in which target patients who meet the registration requirements are randomly allocated to a surgical loupe group and a microscopy group after obtaining their consent (Ratio 1:1).

The procedures used in this study are as follows: A graft from a living donor is transplanted once the liver of the recipient is completely removed. Revascularization is performed followed by biliary reconstruction. The presence of biliary complications is morphologically evaluated by cholangiography under fluoroscopy or magnetic resonance cholangio-pancreatography (MRCP). In addition, computed tomography and postoperative blood tests (increase in liver enzymes and biliary enzymes) are used for evaluation.

## Endpoints

The primary endpoint is an incidence of biliary complications (bile leakage and anastomotic biliary stricture) with Clavien-Dindo class III or higher within 52 weeks following surgery.

Bile leakage was defined as the presence of bile material in a closed suction drain that persisted more than 7 days after transplantation or as the presence of a biloma around the area of the anastomosis. Anastomotic biliary stricture was defined as an intrahepatic biliary dilatation > 3 mm in the presence of a notable anastomotic narrowing, on the basis of symptoms, or on the basis of abnormal liver function tests. Jaundice, fever, or abdominal pain could be present with a bile leak or stricture. The secondary endpoint is length of time required for biliary reconstruction using a surgical microscope.

## Patient eligibility

Patients scheduled to undergo a living donor liver transplantation at Nagasaki University Hospital, Kyusyu University Hospital and University of the Ryukyus Hospital are targeted.

### Inclusion criteria

Patients meeting all of the following criteria are targeted:

1. Recipients of living donor liver transplantation that will be performed in the participating facilities of this study from the date the study is approved
2. Patients who will undergo biliary reconstruction by bile duct to bile duct anastomosis
3. Patients who do not require multiple biliary reconstructions (however, it will not be considered as multiple biliary reconstructions if it forms multiple bile ducts and can be anastomosed as a single hole as a result of bile duct plasty.)
4. Patients who are 18 years of age or older
5. No gender restrictions
6. Patients who voluntarily give their consent to participate in this study upon receiving sufficient explanation and having a good understanding of the details. Patients who are able to provide consent. In addition, regarding patients for whom it is believed difficult to provide effective informed consent, such as when hepatic encephalopathy was caused by hyperammonemia due to the progression of liver failure, consent shall be obtained by their legal representatives. Legal representatives shall include spouses, parents, adult children, adult siblings or grandchildren, grandparents, relatives who share the same household, or individuals believed to be equivalent to those who are closely related.

### Exclusion criteria

1. Patients who have been deemed ineligible as a study subject by the responsible personnel of the study, such as when choledochojejunostomy is performed or when anastomosis under a microscope is difficult.
2. Patients who did not provide their consent to participate in the study upon receiving sufficient explanation regarding their participation in this study.

## Registration

The members and principal investigator of each facility shall receive the consent of the subject patient and contact the Department of Surgery, Nagasaki University Graduate School of Biomedical Sciences by fax or e-mail. The Department of Surgery, Nagasaki University Graduate School of Biomedical Sciences will input UMIN INDICE and allocate it. The allocation results (microscopy group or surgical loupe group) will be provided to the participating facility by fax or e-mail so that each site can perform surgery accordingly.

- Allocation method: minimization
- Allocation factors: type of graft (right liver/left liver), primary disease of the recipient, facility conducting the study
- Method of registering the case: UMIN INDICE
- Institution registering the case and conducting the allocation: The Department of Surgery, Nagasaki University Graduate School of Biomedical Sciences

## Treatment

### Surgical methods

1. Microscopy group

A surgical microscope (5x–15x) is set in the surgical field in order to perform the following procedures: With the diameter of the donor’s bile duct as well as alignment in mind, either the recipient’s left and right bile ducts or common bile ducts are selected as anastomosis points. Interrupted suture with an absorbent suture is performed to suture the back and front of the bile duct walls (size 6–0). All ligations shall be tied outside. A tube is placed in the anastomosis site.

2. Surgical loupe group

Wear a surgical loupe (2.5x–4.5x), which is also used in normal surgery, and perform the following procedures: Set it in the surgical field. With the diameter of the donor’s bile duct as well as alignment in mind, either the recipient’s left and right bile ducts or common bile ducts are selected as anastomosis points. Interrupted suture with an absorbent suture is performed to suture the back and front of the bile duct walls (size 6–0). All ligations shall be tied outside. A tube is placed in the anastomosis part.

## Data collection

This study examines and collects information in accordance with the following schedule. The blood test and abdominal contrast-enhanced CT do not include anything special and is conducted to collect information as in regular medical care.

### Items to collect in the study

- Patient background: gender, age, BMI, primary disease, liver function prior to surgery, surgical history
- Factors of surgery: Completion rate of biliary reconstruction using a surgical microscope, duration of surgery, length of time required for biliary reconstruction
- Blood test: hepatic biliary enzymes (AST, ALT, ALP, γGTP), bilirubin (total bilirubin, direct bilirubin) (***Table 1***)
- Regarding complications: presence of biliary complications: bile leakage (diagnose by drainage and puncture), anastomotic biliary stricture (cases in which treatment such as endoscopic biliary tube insertion or percutaneous cholangiodrainage was required)
- Cholangiographyn (direct imaging, MRCP): evaluate the presence of anastomotic stricture and the presence of anastomotic leakage (***Table 1***).
- Abdominal contrast-enhanced CT: evaluate the presence of intrahepatic bile duct dilatation (***Table 1***).

**Table 1:** Items to collect in the study.


ITEM	PRIOR TO SURGERY	FOLLOWING SURGERY	OBSERVATION PERIOD FOLLOWING SURGERY			UPON DISCONTINUATION

Period	0–4 Weeks before	0 weeks	12 weeks following surgery	24 weeks following surgery	52 weeks following surgery	Upon discontinuation

Medical checkup	○	—	○	○	○	○

Obtainment of consent	○	—	—	—	—	—

Random allocation	•	—	—	—	—	—

Patient background	○	—	—	—	—	—

Factors of surgery (total duration of surgery, length of time required for biliary reconstruction)	—	○	—	—	—	—

Blood test (hepatic biliary enzymes, bilirubin)	○	—	○	○	○	○

Regarding complications	—	—	○	○	○	○

Biliary form evaluation (direct imaging or MRCP)	—	—	○			

Abdominal contrast-enhanced CT	○	—	○	○	○	○


To be conducted upon obtaining consent.

Regarding the postoperative observation period, each evaluation date shall be within the acceptable range of ± 28 days.

## Statistical analysis

The estimated sample size is 158 (79 in each group). As a difference to be detected, the incidence of biliary complications by the conventional method was set to 25%, while the incidence by microscope was set to 10% (the effective rate was calculated as 75% and 90%). The number of cases required was calculated by setting the α error on one side as 5% and the detecting power as 80%. Regarding the biliary reconstruction under a microscope, we will use the Chi-square test to examine whether or not there is a statistically significant difference between the microscopy and the surgical loupe group. The significance level is set to be 5%. When a statistically significant difference is observed between the two, we will determine that biliary reconstructions under a microscope is effective. Exploratory analysis will be conducted for the secondary outcomes.

## Discussion

Biliary complication is more frequent in living donor liver transplantation than deceased donor liver transplantation [5,6,7]. Biliary complication is associated with outcomes of liver transplantation in terms of graft function and quality of life. For anastomotic stricture, an endoscopic approach and percutaneous transhepatic cholangiodrainage (PTCD) are required after surgery, and hepaticojejunostomy may be performed in some cases [9]. Endoscopic management of biliary stricture is technically more challenging in LDLT than in DDLT, because of the complexity of the biliary anastomosis including multiple anastomosis, tortuous and angulated biliary anatomy. Even after the successful endoscopic approach, multiple stent exchange to prevent stent occlusion is needed. In also patients with PTCD, multiple exchange of drainage tube is usually needed. Since quality of life is often disturbed in patients with biliary stricture despite the development of postoperative intervention, prevention of biliary complication is highly important.

For preventing biliary complications, various ideas have been implemented from a surgical point of view. For example, the ischemia to the bile duct stumps, the number and size of bile ducts has been reported as causes of biliary complication. The efficacy of minimal hilar dissection to preserve maximal vascular integrity of the bile duct in recipient surgery [5]. With respect to the number of bile ducts in the graft, we have been performing intraoperative cholangiography with radiopaque filament encircling bile duct to detect the appropriate cut point [10].

As one of the important innovations, biliary reconstruction with a microsurgical technique [11]. This approach has the technical advantage of enhanced visualization of the operative field including biliary reconstruction for conspicuously small bile duct [11]. The technique is considered effective to prevent physical trauma to the bile duct epithelium and conduct more precise placement of stitches during the biliary anastomosis.

According to the report about results of routine microsurgical biliary reconstruction by Lin et al., the incidence of biliary complication significantly decreased compared to the previous reports. In other reports, Yan et al. concluded that microsurgical technique and fixed operator could decrease the biliary complications of LDLT [12]. Favorable outcomes of microsurgical biliary reconstruction have been reported based on single-center experiences.

In the current multi-facility randomized trial, we made a protocol to clarify its efficacy as well as its versatility. To evaluate the responsibility of surgical microscope on postoperative biliary complication, we set type of graft, primary disease of the recipient, facility as allocation factors in this randomized study. All facilities participating in this study have sufficient experiences of living donor liver surgery and microsurgery.

If this study reveals the efficacy and versatility of biliary tract reconstruction with a microscope, it will be one of the options for improving outcomes of biliary reconstruction in living-donor liver transplantation.

## Ethics and Dissemination

This study protocol was approved by the institutional review board of Nagasaki University Hospital (No. 20122102-2). The study is registered in UMIN-CTR as UMIN000042011. Written informed consent will be obtained from all participants. The results will be published in a peer-reviewed journal and will be presented at medical meetings.
